# Fructose-1,6-bisphosphatase deficiency: estimation of prevalence in the Chinese population and analysis of genotype-phenotype association

**DOI:** 10.3389/fgene.2024.1296797

**Published:** 2024-07-05

**Authors:** Qi Ni, Meiling Tang, Xiang Chen, Yulan Lu, Bingbing Wu, Huijun Wang, Wenhao Zhou, Xinran Dong

**Affiliations:** ^1^ Children’s Hospital and Institutes of Biomedical Sciences, Fudan University, National Children’s Medical Center, Shanghai, China; ^2^ Center for Molecular Medicine, Children’s Hospital of Fudan University, National Children’s Medical Center, Shanghai, China; ^3^ Division of Neonatology, Children’s Hospital of Fudan University, National Children’s Medical Center, Shanghai, China; ^4^ Guangzhou Women and Children's Medical Center, Guangzhou Medical University, Guangzhou, Guangdong, China

**Keywords:** fructose-1, 6-bisphosphatase deficiency, prevalence estimation, curation for pathogenic variants, newborn screening, allele frequency comparison, genotypephenotype analysis

## Abstract

**Objective:**

Fructose-1,6-bisphosphatase deficiency (FBP1D) is a rare inborn error due to mutations in the *FBP1* gene. The genetic spectrum of FBP1D in China is unknown, also nonspecific manifestations confuse disease diagnosis. We systematically estimated the FBP1D prevalence in Chinese and explored genotype-phenotype association.

**Methods:**

We collected 101 *FBP1* variants from our cohort and public resources, and manually curated pathogenicity of these variants. Ninety-seven pathogenic or likely pathogenic variants were used in our cohort to estimate Chinese FBP1D prevalence by three methods: 1) carrier frequency, 2) permutation and combination, 3) Bayesian framework. Allele frequencies (AFs) of these variants in our cohort, China Metabolic Analytics Project (ChinaMAP) and gnomAD were compared to reveal the different hotspots in Chinese and other populations. Clinical and genetic information of 122 FBP1D patients from our cohort and published literature were collected to analyze the genotype-phenotypes association. Phenotypes of 68 hereditary fructose intolerance (HFI) patients from our previous study were used to compare the phenotypic differences between these two fructose metabolism diseases.

**Results:**

The estimated Chinese FBP1D prevalence was 1/1,310,034. In the Chinese population, c.490G>A and c.355G>A had significantly higher AFs than in the non-Finland European population, and c.841G>A had significantly lower AF value than in the South Asian population (all *p* values < 0.05). The genotype-phenotype association analyses showed that patients carrying homozygous c.841G>A were more likely to present increased urinary glycerol, carrying two CNVs (especially homozygous exon1 deletion) were often with hepatic steatosis, carrying compound heterozygous variants were usually with lethargy, and carrying homozygous variants were usually with ketosis and hepatic steatosis (all *p* values < 0.05). By comparing to phenotypes of HFI patients, FBP1D patients were more likely to present hypoglycemia, metabolic acidosis, and seizures (all *p*-value < 0.05).

**Conclusion:**

The prevalence of FBP1D in the Chinese population is extremely low. Genetic sequencing could effectively help to diagnose FBP1D.

## Introduction

Fructose-1,6-bisphosphatase (FBPase) deficiency (FBP1D, OMIM: 229700 OMIM, 2021) is an inherited autosomal recessive (AR) disease caused by the *FBP1* gene mutations, which leads to the FBPase reduction or deficiency (Bhai et al., 2018; Zahoor et al., 2020). FBPase is a key enzyme of gluconeogenesis, and its reduction significantly reduce the endogenous formation of glucose from the available precursor, such as pyruvate, lactate, glycerol and alanine (gluconeogenic amino acids) (Emecen Sanli et al., 2022). This is because that the FBPase inactivation impairs liver formation of glucose from all gluconeogenic precursors (Tran, 2017). FBP1D is also considered as an inborn error in the pathway of fructose metabolism (Tang et al., 2022). In most cases, the main clinical manifestations are ketonic hypoglycemia, hyperlactacidemia and metabolic acidosis (Gorce et al., 2022). Other phenotypes include vomiting after eating fructose-related foods, epileptic seizure and impaired liver function displayed as elevated hepatic transaminase, hyperbilirubinemia, hepatomegaly, and hepatic steatosis. Severe phenotypes included reduced consciousness, coma, dyspnea, and death (often due to delayed diagnosis) (Moey et al., 2018). Neurological complications were reported with inappropriate treatment (Hasegawa et al., 2003). Although no specific treatment for FBP1D nowadays, long-term prognosis is pretty good with early diagnosis and avoidance of hunger and infection, as well as dietary fructose restriction (Tran, 2017).

The prevalence of FBP1D is approximately 1/350,000 in Netherlands, 1/900,000 in France (Cao and Pan, 2022), 1/147,575 in Italy and 1/1,782,321 in Southern Brazilian (Pinheiro et al., 2019). In the America, the patients are estimated to be fewer than 5,000(Center, Accessed 01 November 2022). The FBP1D prevalence in the Chinese population remain unknown (Yi and Xie, 2022).

After literature review, we found different population had its own hotspots. For example, two missense mutations c.841G>A and c.472C>T are reported as the most common alleles in the India and Pakistan population (Bhai et al., 2018; Zahoor et al., 2020), c.958G>A and c.986T>C are the most frequent variants in the Southern Brazilian (Pinheiro et al., 2019), while c.114_119dup is prevalent in Saudi Arabia (Salih et al., 2020), c.960_961insG and c.490G>A are common in Japan (Kikawa et al., 1997; Hasegawa et al., 2003). The situation for the Turkish patients is significantly different, as the exon one deletion (NM_000507.4) is the most common variant and considered as the founder mutation in Turkish FBP1D patients. The hotspots in the Chinese population are unexplored.

To date, the integration of genotype and phenotype can lead to a more precise pathway of diagnosis and therapy (Dong et al., 2022), however the relationship between genotype-phenotype in FBP1D patients keep ambiguous. Kilic et al. studied 10 patients from nine families with FBP1D but no significant genotype and phenotype correlation was found (Kilic et al., 2019). One of the largest case report cohorts is Hasegawa Y, et al., which retrospectively analyzed phenotypes of 20 FBP1D patients (Hasegawa et al., 2003). They did not identify genotype and phenotype associations either. Previous studies have generally reported that the severity of the disease depend on the feeding strategy and infection condition of the individual (Tran, 2017; Kilic et al., 2019). The genotype-phenotype correlation is still confusing due to the small sample size of previously reported cohorts, since most of the variants are sporadic, and the clinical manifestations and laboratory findings are heterogeneous.

In this study, we collected *FBP1* variants and FBP1D patients from our Chinese Children’s Rare Disease Genetic Testing Clinical Collaboration System (CCGT) and public resources to reveal genotypes, phenotypes and their relationship in Chinese and other populations. Furthermore, we compared the clinical phenotypes between FBP1D and another inborn error of fructose metabolism hereditary fructose intolerance (HFI, OMIM: 229600 GARD, 2022). Our results gave a clue to the genotype-phenotype association of FBP1D.

## Methods

### Data acquisition for CCGT

This study was approved by the Ethics Committee (2022–215) of Fudan University Children’s Hospital. Informed consent was signed by the patient’s parents in the clinic or ward. The study was conducted in accordance with the guidelines of the Helsinki declaration. CCGT is one of the largest exome genetic databases of the Chinese population, which consists of children suspected of having genetic diseases and their parents. CCGT is an internal database and divides into children cohort (CCGT_C) and parents cohort (CCGT_P). No consanguineous couples were identified within the CCGT_P.

The CCGT cohort was the same as in our previous studies, and the processing steps can be found in those studies (Ni et al., 2022; Tang et al., 2022). In brief, we consulted and obtained the informed consent of the patient’s parents. Everyone received WES or clinical exome sequencing (CES), both of which covered the exon region and exon–intron splicing junction region (deep intron to 15 bp) of *FBP1* genes. Both tests were sequenced on the Illumina HiSeq X10 with 150 bp paired-end sequencing. Genetic diagnosis of FBP1D is conducted by experienced clinicians and genetic consultants according to ACMG guidelines. For the prevalence estimation and Allele frequency (AF) comparison of FBP1D, we excluded the diagnosed children from CCGT_C. As none of their parents ever took an ES testing, no individual was excluded from CCGT_P.

### Literature search of FBP1D-related studies

PubMed, Web of Science and China national knowledge infrastructure (CNKI) were searched using the terms “fructose-1,6-bisphosphatase deficiency”, “fructose-1,6-bisphosphatase deficiency and case report”, “*FBP1* mutation”, and “*FBP1* variant” between 1970 (first described) and July 2022 (Emecen Sanli et al., 2022). We applied strict literature inclusion criteria to make a more accurate conclusion. Our literature inclusion criteria were as follows: 1) the literature reported cases of FBP1D; 2) the nomenclature of variants met the requirements of HGVS (den Dunnen et al., 2016); 3) the variants were evaluated as P/LP according to ACMG guidelines (Richards et al., 2015; Abou Tayoun et al., 2018); and 4) the literature represented high-quality studies. The exclusion criteria were as follows: 1) lack of information on genotype or phenotype; 2) repeated cases. According to those criteria, a total of 729 articles were found, of which 31 were finally included in this study (Kikawa et al., 1995; Kikawa et al., 1997; Herzog et al., 1999; Beatty et al., 2000; Matsuura et al., 2002; Hasegawa et al., 2003; Tavil and Sipahi, 2003; Faiyaz-Ul-Haque et al., 2009; Asberg et al., 2010; Moon et al., 2011; Afroze et al., 2013; Eren et al., 2013; Kamate et al., 2014; Kato et al., 2015; Lebigot et al., 2015; Li et al., 2017; Bhai et al., 2018; Moey et al., 2018; Sharma et al., 2018; Kilic et al., 2019; Lee et al., 2019; Pinheiro et al., 2019; Mahmut et al., 2020; Salih et al., 2020; Wang et al., 2020; Zahoor et al., 2020; Devaney et al., 2021; Pinheiro et al., 2021; Samprathi et al., 2021; Cao and Pan, 2022; Emecen Sanli et al., 2022) and 117 published FBP1D patients were collected for genotype-phenotype association studies.

### Curation of P/LP variants in the *FBP1* gene

We included reported variants of the *FBP1* gene from Human Gene Mutation Database (HGMD, level DM/DM?), ClinVar (level P/LP), and the FBP1D-related literature mentioned above. No new mutation sites were detected in our cohorts. All mutation sites were curated by at least two experienced geneticists. After manually checking, 97 out of the 101 variants were curated at the P/LP level.

### Estimation of FBP1D prevalence in the Chinese population

We estimated the FBP1D prevalence by three strategies as described in our previous studies (Ni et al., 2022; Tang et al., 2022). The first method was based on the carrier frequencies of individuals in CCGT_C and CCGT_P, Hardy-Weinberg principle is used to calculate the prevalence. A carrier frequency *p* of all P/LP variants for *FBP1* could be obtained in each cohort. The risk for a FBP1D child was calculated as 1/4*p ^2^. Then second method was based on the principle of permutation and combination in mathematics. The carrier frequency in male *p*_m_ and female *p*_f_ were calculated in each cohort, then the risk for a FBP1D child was calculated as 1/4* *p*_m_ * *p*_f_. In this strategy, the probability of affected children is calculated by randomly selecting male individuals carrying the P/LP FBP1 variants and female individuals also carrying the P/LP *FBP1* variants. The third method was based on the Bayesian framework with 95% confidence interval given according to (Schrodi et al., 2015). The posterior mean value of FBP1D affected probability was estimated as the second moment of posterior Beta distribution by the following equation (α = x, β = 2n-x) where x is the number of alleles with P/LP variants and n is the number of individuals in the cohort.

$${\left(\frac{\alpha }{\alpha +\beta }\right)}^{2}+\frac{\alpha \beta }{{\left(\alpha +\beta \right)}^{2}\left(\alpha +\beta +1\right)}$$

### Collection of public FBP1D variant frequency

China Metabolic Analytics Project (ChinaMAP) was introduced as an external database of Chinese population. It is based on cohort studies across diverse regions and ethnic groups with metabolic phenotypic data in China, and analysis of the whole genome sequencing data in 10,588 healthy individuals (Cao et al., 2020). It is one of the biggest public Chinese population data we can access now.

The AF of the *FBP1* gene in other populations were obtained from the gnomAD (Karczewski et al., 2020). The gnomAD database collects sequencing data of healthy individuals from various races. South Asian, East Asian, African American, Ashkenazi Jewish, admixed American, Finnish in Finland, and non-Finland European populations in gnomAD were included.

### Data acquisition for studies of genotype-phenotype relationships and comparison with HFI

Common phenotypes of FBP1D and HFI were figured out from the HPO project (Köhler et al., 2021) and OMIM. To compare with FBP1D, elevated hepatic transaminase was divided from abnormal liver function tests and neonatal hyperbilirubinemia was divided from jaundice in HFI common phenotypes. We recorded the genotype and whether they had the common phenotypes of the 122 FBP1D patients from the CCGT and the published literature mentioned above. The genotypes were further grouped by variants’ mutation types and zygosities. Frequencies of HFI common phenotypes were obtained from 68 HFI patients collected in our previous study (Tang et al., 2022).

### Statistical analyses

All statistical analysis was performed by R version 4.0.3. Chi-square test (λ2. test) was used by default. When the conditions are not met, Fisher’s exact test was used. *p* values were adjusted by “fdr” strategy for multiple tests.

## Results

### Curation of *FBP1* pathogenic variants and allele frequency analysis

**FIGURE 1:**
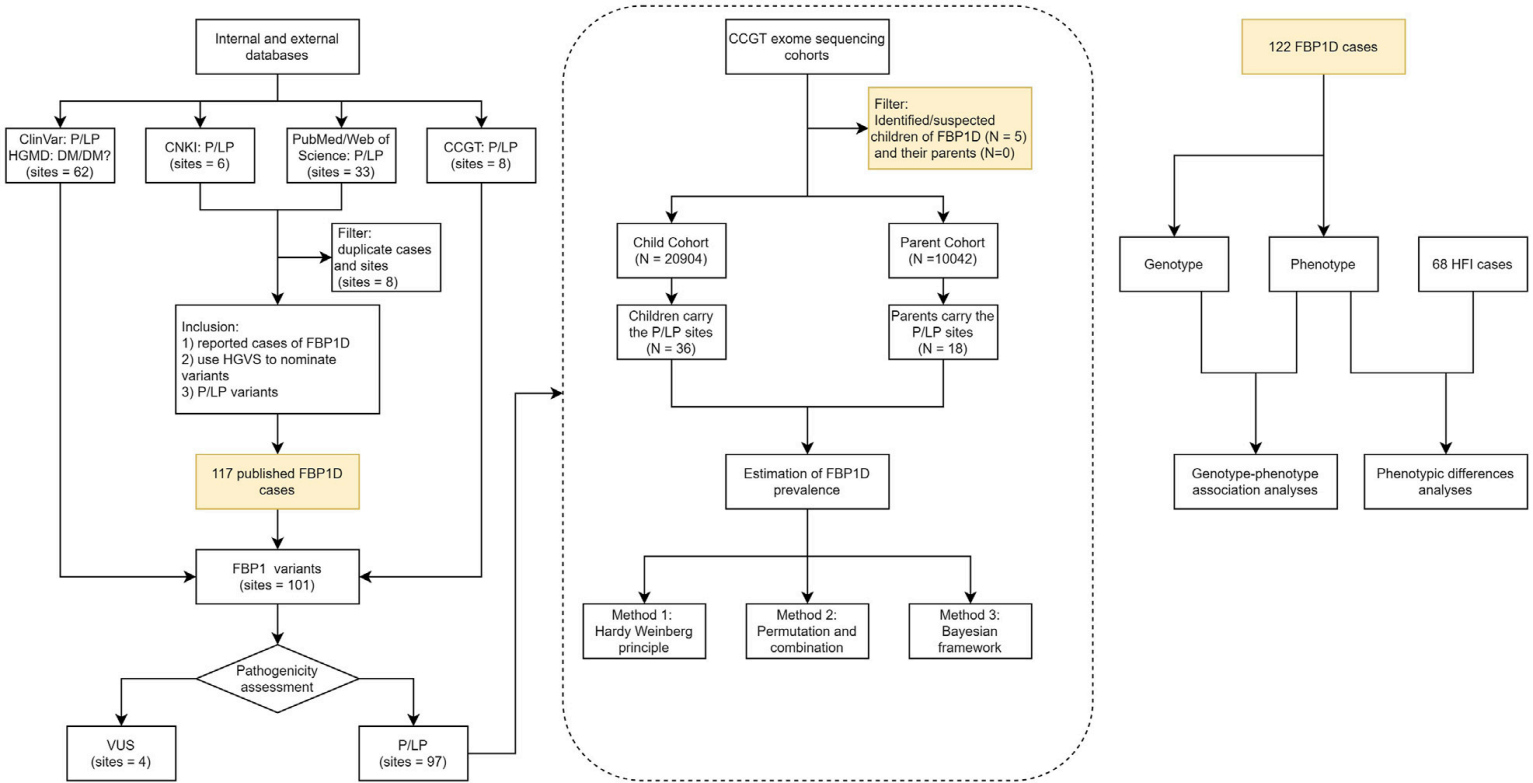
The workflow for estimation of FBP1D prevalence in the Chinese population. This study consisted of three parts: (1) curation of *FBP1* variants pathogenicity. (2) estimation of Chinese FBP1D prevalence. (3) association analysis of genotype-phenotype.

**FIGURE 2:**
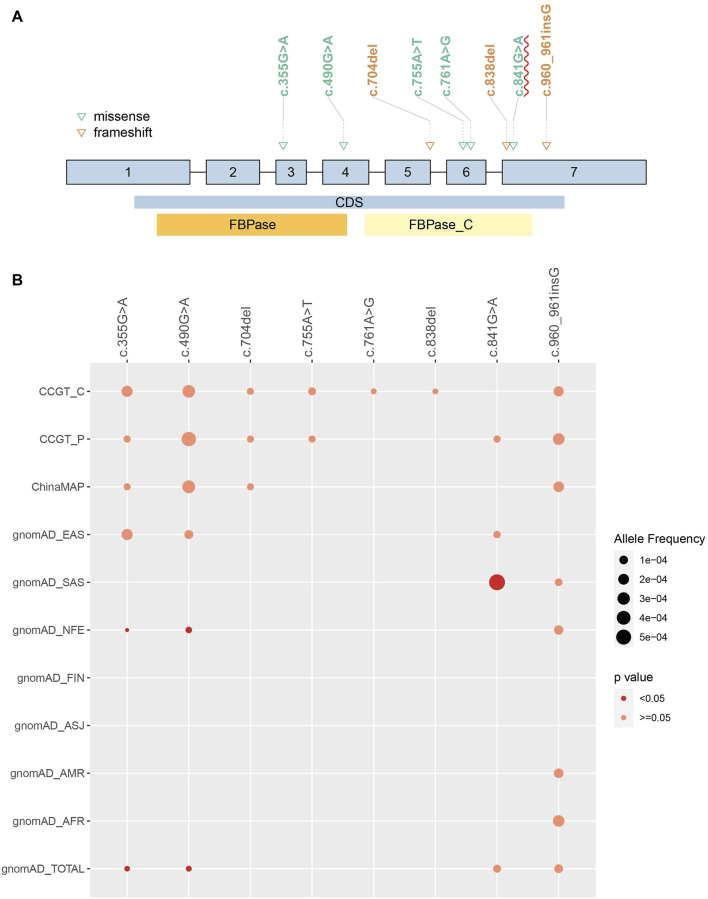
Allele frequency of observed pathogenic variants in Chinese population and comparison to other populations. Eight pathogenic FBP1 variants were observed in Chinese population. **(A)** Eight variants position on FBP1 gene. Patients carrying homozygous c.841G>A were more likely to present increased urinary glycerol than without these variants (*p*-value = 0.010). CDS: coding sequence. FBPase and FBPase_C: two domains of FBP1 protein. **(B)** AF of each variant in different populations were compared to CCGT_C cohort. The size of the dot represented AF. The red color represented significantly different AF compared to CCGT_C. No dot means that variant has not been detected in the population. AFR: African American; ASJ: Ashkenazi Jewish; NFE: non-Finland European population; FIN: Finnish in Finland; AMR: admixed American population; SAS: South Asian; EAS: East Asian population.

After pathogenicity assessment of *FBP1* variants collected from the CCGT, HGMD, ClinVar, CNKI, Web of Science, and PubMed, 97 P/LP variants were identified, including frameshift (35%, 34/97), missense (32%, 31/97), splicing (15%, 15/97), nonsense (10%, 10/97), CNV (5%, 5/97) and in-frame indel (2%, 2/97) variants (Figure 1; Supplementary Table S1). To compare AF of these variants in Chinese population with other populations, we divided CCGT into two unrelated sub cohorts, CCGT_C and CCGT_P. Then, we excluded children diagnosed with FBP1D from CCGT_C and their parents from CCGT_P. Among the 97 P/LP variants, 38 have been documented in the CCGT, ChinaMAP and gnomAD (Supplementary Table S2). Eight of the 38 variants were observed in Chinese (Figure 2). Of the eight variants, c.490G>A and c.355G>A had significantly higher AF in the Chinese population than in the non-East Asian populations, while c.841G>A had significantly lower AF value in the Chinese population than in the South Asian population (all *p* values < 0.05). In addition, c.490G>A is the most common variant in the Chinese population (AF in CCGT_C: 1/3216, in CCGT_P: 1/2232; and in ChinaMAP: 1/3025), while it has not been reported in African American, admixed American, Ashkenazi Jewish, Finnish in Finland and South Asian populations.

### Estimation of FBP1D prevalence in the Chinese population

We estimated the FBP1D prevalence based on 20,904 pediatric patients in the CCGT_C cohort (12,771 males and 8,133 females) and 10,042 parental samples in the CCGT_P cohort (5,014 males and 5,028 females). The total number of individuals carrying P/LP *FBP1* variants was 36 (25 males and 11 females) in the CCGT_C and 18 (11 males and seven females) in the CCGT_P. Based on the carrier frequency, the estimated FBP1D prevalence were 1/1,348,695 in the CCGT_C and 1/1,244,960 in the CCGT_P. By the permutation and combination method, the estimated prevalence were 1/1,510,786 and 1/1,179,494 respectively. By the Bayesian framework, the estimated prevalence of FBP1D were 1/1,312,275 (95% confidence interval was 1/2,748,617∼1/737,905) in the CCGT_C and 1/1,179,494 (95% confidence interval was 1/3,542,901∼1/544,684) in the CCGT_P. In general, the estimated prevalence of FBP1D in the Chinese population is 1/1,310,034 by averaging all the above results (Table 1).

**TABLE 1:** FBP1D prevalence estimation in CCGT children cohort and parents cohort with estimated affected frequency by three methods.

	Children cohort	Parents cohort
Total number	20904	10042
Gender (Female/Male)	8133/12771	5028/5014
Carrier with P/LP variants (Female/Male)	11/25	7/11
Carrier frequency	1/580	1/558
Method 1: carrier frequency	1/1348695	1/1244960
Method2: permutation and combination	1/1510786	1/1309631
Method 3: Bayesian framework (95% CI)	1/1312275 (1/2748617∼1/737905)	1/1179494 (1/3542901∼1/544684)
Average	1/1310034	

### Genotype and phenotype of six Chinese FBP1D patients

Five patients were diagnosed as FBP1D in the CCGT_C. Another published FBP1D patient diagnosed in our center from other cohort was also collected in this study by literature review (Wang et al., 2020). Here we add detailed clinical phenotypes and prognosis of this patient (Table 2; Supplementary Table S3). Of these six patients, two presented phenotypes in the neonatal period, two in their early infancy (<1 year) and two in childhood. Hypoglycemic episodes was the first symptom for most patients (5/6, Table 2), mostly associated with fever or infection (4/5). Two children presented with nervous system symptoms, and their prognosis was not very optimistic, and even one child died. Based on clear genetic test results and typical clinical manifestations, all patients were diagnosed with FBP1D. After early dietary guidance (avoid feeding the fructose-related foods and avoid prolonged fasting) and avoiding infection or other triggering factors, most patients (4/6) did not present poor prognoses and developed well.

**TABLE 2:** Genotype and clinical manifestations of six Chinese FBP1D patients.

ID	Mutations	Gender	Age (years)	Age of onset	Phenotypes at onset	Prognosis	AF in CCGT_C	AF in gnomAD
1	c.355G>A (paternal), c.960_961insG (maternal)	Male	—	1 year and 8 months	Status epilepticus, vomiting, severe infection with fever	Death	2.15^*^10^−4^ 1.67^*^10^−4^	1.99^*^10^−5^ 1.03^*^10^−4^
2	c.490G>A (paternal), c.490G>A (maternal)	Female	7	3 years	Convulsions, unconsciousness, hypoglycemia, fever	With brief epileptic seizure	3.11^*^10^−4^	2.40^*^10^−5^
3	c.841G>A (paternal), c.778G>A (maternal)	Male	6	11 months	Rotavirus infection, hypoglycemia, metabolic acidosis, hepatomegaly	Without symptoms for 2 years	<2.39^*^10^−5^ <2.39^*^10^−5^	7.56^*^10^−5^ 7.96^*^10^−6^
4	c.355G>A, exon1 deletion	Male	9	Neonatal	Episodic tachypnea, hypoglycaemic episodes, pneumonia	Occasional hypoglycemia	2.15^*^10^−4^ <2.39^*^10^−5^	1.99^*^10^−5^ 9.22^*^10^−5^
5	c.704del (paternal), c.959dup (maternal)	Male	8	Neonatal	Vomiting, hypoglycemia, hyperbilirubinemia	Occasional nighttime hypoglycemic attacks	4.78^*^10^−5^ <2.39^*^10^−5^	0 0
6^a^	c.333 + 2T>G (paternal), c.333 + 2T>G (maternal)	Male	6	7 months	Hypoglycemia, lactic acidosis, metabolic acidosis, fever	Occasional vomiting and hypoglycemia	<2.39^*^10^−5^	0

^*^ This patient has been reported in Wang’s study (Wang et al., 2020). The transcript used for the entire article is NM_000507.4. To accurately estimate the prevalence of FBP1D, we have excluded these patients in CCGT_C. No individul carried c.841G>A, c.778G>A, exon1 deletion, c.959dup and c.333 + 2T>G in the filtered CCGT_C, so the AF, of these variants were displayed as <2.39*10−5. AF: allele frequency.

### Analysis of genotype-phenotype relationship in 122 FBP1D patients

We collected genotype and phenotype of 122 FBP1D patients, including the six patients mentioned above and other 116 patients from literature review (Supplementary Table S3). The most common clinical phenotype was hypoglycemia (111/113, 98.2%), and then was metabolic acidosis (102/121, 84.3%). Other common phenotypes included vomiting (64/107, 59.8%), ketosis (61/83, 73%), hepatomegaly (54/89, 61%), fever (49/94, 52%), respiratory distress (30/51, 59%) and seizures (27/72, 37%). When comparing combinations of variants, patients carrying homozygous exon1 deletion were more likely to present hepatic steatosis than without these variants (OR = 5.4, *p*-value = 0.028), while carrying homozygous c.841G>A were more likely to present increased urinary glycerol than without these variants (*p*-value = 0.010) (Figure 2A; Supplementary Table S4. Mutation types of all variants in the 122 FBP1D patients were classified into missense, nonsense, splicing, frameshift, in-frame indel and CNV (Supplementary Table S5). Two missense variants were the most common mutation type combination in FBP1D patients (48/122, 39.3%), then were two frameshift variants (22/122, 18.0%) and two CNVs (15/122, 12.3%). For mutation type-phenotype analyses, we found that patients carrying two CNVs were more likely to present hepatic steatosis (OR = 17.9, *p*-value = 0.009). For zygosity-phenotype analyses, patients carrying compound heterozygous pathogenic variants were more likely to present lethargy (*p*-value = 0.040), and patients carrying homozygous pathogenic variants were more likely to present ketosis (*p*-value = 0.007) and hepatic steatosis (*p*-value = 0.015) (Supplementary Table S6).

### Comparison of clinical manifestations between FBP1D and HFI

**FIGURE 3:**
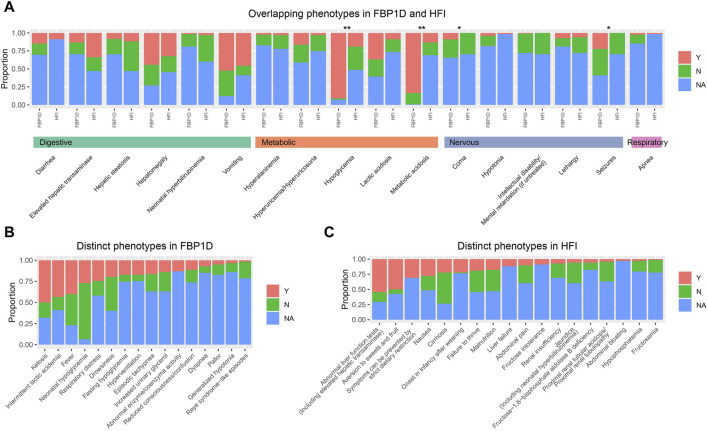
Comparison of phenotypes between FBP1D and HFI. Proportion of patients with/without phenotype in **(A)** Overlapping phenotypes in FBP1D and HFI. **(B)** Distinct phenotypes in FBP1D. **(C)** Distinct phenotypes in HFI. Y: patients with the phenotype; N: patients withou the phenotype; NA: patients did not mention about the phenotype. **: adjusted *p*-value ≤0.001; *: adjusted *p*-value <0.05.

HFI is also a severe inborn errors of fructose metabolism caused by mutations in *ALDOB* gene. We extracted 33 common clinical phenotypes of FBP1D and 35 common clinical phenotypes of HFI from OMIM and HPO. Then we thoroughly calculated the frequency of these phenotypes in the 122 collected FBP1D patients and 68 HFI patients from our previous study (Tang, et al., 2022). Seventeen phenotypes are overlapped in these two diseases (Figure 3A; Supplementary Table S7), while FBP1D has 16 relatively distinct phenotypes and HFI has 18 relatively distinct phenotypes (Figure 3B, C; Supplementary Table S8). Though FBP1D and HFI are both metabolism disorders, FBP1D patients markedly have hypoglycemia and metabolic acidosis than HFI patients (OR = 88 and seven respectively, all *p*-value < 0.05). Besides, FBP1D patients more probably have seizures and coma than HFI patients (all *p*-value < 0.05). Interestingly, we found that HFI patients are more likely to present diarrhea than FBP1D patients, though the difference was not significant after statistical adjustment. Comparing to HFI, fever and ketosis are considered as relatively distinctive phenotypes of FBP1D.

## Discussion

In this study, we collected and curated 97 P/LP variants of *FBP1*. Similar with Japanese population, c.490G>A and c.960_961insG are common in Chinese, besides c.355G>A has significantly higher AF in Chinese compare with non-Finland European populations. The most frequent P/LP *FBP1* variant of Chinese is c.490G>A, while the missense variant c.841G>A is the most common in South Asian and the CNV exon1 deletion is the most common in Turkish population (Kilic et al., 2019). Nonetheless, many FBP1D patients carry sporadic novel variants. For example, Lebigot et al. reported a Greece patient with homozygous c.865dupA variant (Lebigot et al., 2015). None carrier of this variant has been observed in gnomAD, CCGT or ChinaMAP. Pinheiro et al. reported two novel variants found in Brazilian patients: c.958G>A and c.986T>C (Pinheiro et al., 2019). None of these variants has been recorded in gnomAD, CCGT or ChinaMAP either. This may due to the conservation of *FBP1*. The observed/expected (oe) value of *FBP1* loss-of-function variants from gnomAD is 0.32 (90% CI: 0.15–0.75), lower than *ALDOB* (0.68 and 90% CI is 0.43–1.1), indicating *FBP1* is under stronger selection than *ALDOB*. *FBP1* may involve more pathways than gluconeogenesis and more studies needed. Considering the conservation of *FBP1* and the high frequency of novel variants on it, sequencing the entire gene would be a better way to reduce missed diagnosis rate when patients are suspected with FBP1D.

The estimated prevalence of FBP1D in the Chinese population was extremely low, as 1/1,310,034 in our cohort compared with 1/350,000 in Netherlands and 1/900,000 in France (Lebigot et al., 2015). Before our study, there was no reported prevalence for FBP1D in China. To date, only twelve Chinese patients have been documented (Supplementary Table S3), while the total population of China was 1.4 billion (NBSC, 2024), which also supports the inference of a notably low prevalence of FBP1D in China. However, the occurrence of FBP1D patients in our cohort was considerably high (FBP1D patients in CCGT_C: 1/4182 vs. estimated prevalence: 1/1310034). Since gluconeogenesis is activated after a moderate period of fasting in adults but more quickly in newborns or young children, who do not have enough glycogen stocks to provide their glucose needs through glycogenolysis only, most patients appear manifestations in their early age, and usually from the first day of life. As one of the largest children hospital in China, undiagnosed patients, especially neonates, with complicated phenotypes were often sent to our hospital, causing the accumulation of rare disease patients. To avoid bias in carrier analysis, we excluded FBP1D patients and their relatives from our study cohorts to estimate prevalence.

We collected 122 FBP1D patients from literature review and the CCGT database, and analyzed the relationship between clinical phenotypes and genotypes, including variant sites, mutation types, and zygosity. Metabolic abnormalities were the chief symptoms in the FBP1D patients, additional symptoms involved digestive, respiratory and nervous system. Missense variant was the most common mutation type in FBP1D patients. Patients carrying homozygous exon1 deletion were more likely to present hepatic steatosis. Disruption of the *fbp1* gene in mice alters liver metabolic homeostasis and supports tumorigenesis, and eventually develop hepatomegaly and steatosis. Similar mechanisms may operate in FBP1D patients due to metabolic stress (Gorce et al., 2022).

Previous studies have shown that the differential diagnoses between FBP1D and HFI could be very difficult according to clinical manifestations, but the tolerance to fructose in FBP1D is higher than in patients with HFI (Tran, 2017). In our study, we found that the FBP1D patients more likely manifest hypoglycemia, metabolic acidosis and seizures than HFI patients. Besides, fever is a typical trigger for FBP1D comparing to HFI. FBPsae is not only an enzyme in the fructose metabolism pathway, but also a key enzyme in the gluconeogenesis pathway. Pathogenic *FBP1* gene mutations impair FBPase activity, causing patients unable to effectively metabolize pyruvate and lactate to glucose for energy demand during fasting or infection, leading to hypoglycemia and metabolic acidosis (Emecen Sanli et al., 2022). Long-term hypoglycemia can cause hypoglycemic encephalopathy, thereby showing symptoms of seizures. The different functions of FBPase and aldolase B lead to the different clinical manifestations between the two diseases. Genetic testing of variants in genes is a practical method for differential diagnosis.

### Limitations of the study

Our results gave a clue to the genotype-phenotype association of FBP1D. However, most published cases only reported the chief symptoms and do not mention whether they have other symptoms, introducing much missing values for genotype-phenotype analysis and thus result in non-significant results. More comprehend records of patients, especially whether they do not have some phenotypes, would help to clarify the genotype-phenotype relationship.

## Conclusion

The prevalence of FBP1D is extremely low in China, c.490G>A and c.355G>A were hotspots of *FBP1* in Chinese. Hypoglycemia is a typical symptom in FBP1D patients, additional remarkable symptoms including hepatomegaly, fever, respiratory distress and seizures. Genetic sequencing is an effective method for diagnosis of FBP1D.

## Data Availability

The data presented in the study are deposited in the PICOTEES repository at https://birthdefectlab.cn:3000/, where registration is required for access.

## Abbreviations

FBP1D: fructose-1,6-bisphosphatase deficiency; FBPase: fructose-1,6-bisphosphatase; AR: autosomal recessive; CCGT: Chinese Children’s Rare Disease Genetic Testing Clinical Collaboration System; AF: allele frequency; HGMD: Human Gene Mutation Database; HFI: hereditary fructose intolerance; CNKI: China National Knowledge Infrastructure; CES: clinical exome sequencing; WES: whole-exome sequencing; P/LP: pathogenic/likely pathogenic; VUS: variant of unknown significance; ChinaMAP: China Metabolic Analytics Project; HPO: Human Phenotype Ontology; oe: observed/expected.
